# The DREADD agonist clozapine *N*-oxide (CNO) is reverse-metabolized to clozapine and produces clozapine-like interoceptive stimulus effects in rats and mice

**DOI:** 10.1038/s41598-018-22116-z

**Published:** 2018-03-01

**Authors:** Daniel F. Manvich, Kevin A. Webster, Stephanie L. Foster, Martilias S. Farrell, James C. Ritchie, Joseph H. Porter, David Weinshenker

**Affiliations:** 1 Department of Human Genetics, Emory University School of Medicine, Atlanta, GA 30322 USA; 2 Department of Psychology, Virginia Commonwealth University, Richmond, VA 23284 USA; 3 Department of Genetics, University of North Carolina at Chapel Hill School of Medicine, Chapel Hill, NC 27514 USA; 4 Department of Pathology and Laboratory Medicine, Emory University School of Medicine, Atlanta, GA 30322 USA

## Abstract

Clozapine-N-oxide (CNO) has long been the ligand of choice for selectively activating Designer Receptors Exclusively Activated by Designer Drugs (DREADDs). However, recent studies have challenged the long-held assertion that CNO is otherwise pharmacologically inert. The present study aimed to 1) determine whether CNO is reverse-metabolized to its parent compound clozapine in mice (as has recently been reported in rats), and 2) determine whether CNO exerts clozapine-like interoceptive stimulus effects in rats and/or mice. Following administration of 10.0 mg/kg CNO, pharmacokinetic analyses replicated recent reports of back-conversion to clozapine in rats and revealed that this phenomenon also occurs in mice. In rats and mice trained to discriminate 1.25 mg/kg clozapine from vehicle, CNO (1.0–20.0 mg/kg) produced partial substitution for the clozapine stimulus on average, with full substitution being detected in some individual animals of both species at doses frequently used to activate DREADDs. The present demonstration that CNO is converted to clozapine and exerts clozapine-like behavioral effects in both mice and rats further emphasizes the need for appropriate control groups in studies employing DREADDs, and highlights the utility of the drug discrimination procedure as a tool with which to screen the off-target effects of novel DREADD agonists.

## Introduction

Designer Receptors Exclusively Activated by Designer Drugs (DREADDs), a series of engineered human muscarinic receptors that respond exclusively to the synthetic ligand clozapine *N*-oxide (CNO)^1^, have emerged as a popular tool among neuroscience researchers. When DREADDs are expressed in targeted subpopulations of neurons *in vivo*, the activity of those neurons can be manipulated via administration of CNO, providing a powerful technique with which to dissect the neural circuitry underlying complex biological processes and behaviors^2^. The functionality of DREADDs rests upon the assumption that CNO itself is an inert compound that lacks pharmacological activity *in vivo* at non-DREADD targets, but this notion has recently been called into question. It has been reported that CNO can bind to non-DREADD receptors at concentrations required for DREADD activation^3^, and undergoes reverse-metabolism to its parent compound clozapine, an atypical antipsychotic that acts at a variety of pharmacological targets and produces numerous physiological and behavioral effects. The reverse-metabolism of CNO to clozapine has been previously demonstrated in several mammalian species including human^4,5^, monkey^6^, guinea pig^5^, and rat^7,8^. However, to the best of our knowledge, whether this pharmacokinetic conversion also occurs in the mouse (the species most commonly employed in DREADD-based studies) has only been directly tested once^9^. The authors concluded that the emergence of clozapine following CNO administration in mice occurred at insignificant quantities, and that study is routinely cited in murine DREADD papers as evidence against the occurrence of reverse-metabolism. However, back-conversion of CNO to clozapine has now been detected in blood in rats^8^, and a new study found that clozapine, but not CNO, can cross the blood-brain barrier and activate DREADDs following CNO administration in rats and mice^3^. These recent findings would thus suggest that the reverse-metabolism of CNO to clozapine may also be an important determinant of CNO-induced activation of DREADDs in the mouse, but pharmacokinetic data in support of this conclusion are lacking. Perhaps an even more serious consequence of this potential reverse-metabolism is that the converted clozapine may exert activity at endogenous non-DREADD targets, resulting in pharmacological effects that could confound findings derived from studies employing DREADDs. While a recent study in rats reported that CNO can disrupt some behavioral and neurochemical measures in the absence of DREADDs, not all behaviors assayed were modulated by CNO^8^. Moreover, the off-target effects of CNO were not investigated in mice.

In light of these collective findings, we sought to rigorously assess whether CNO is pharmacokinetically converted to clozapine in mice, and secondarily, to determine whether CNO administration produces clozapine-like physiological or behavioral effects in rats and/or mice that lack DREADD expression. To the latter aim, rather than test the effects of CNO across a battery of behavioral and physiological assessments previously reported to be sensitive to clozapine, we instead chose to employ the drug discrimination procedure, a singular operant-behavioral assay in which animals are trained to use the interoceptive (i.e. “subjective”) drug state as a discriminative cue to guide response allocation to one of two levers that is reinforced with food presentation. During training, one lever is reinforced when a drug (e.g., clozapine) has been administered prior to the session, while the alternative lever is reinforced if the drug’s vehicle has been administered prior to the session. Thus, the animals learn to respond on the clozapine-appropriate lever when the interoceptive stimulus effects of clozapine are present, and the vehicle-appropriate lever when clozapine’s interoceptive stimulus effects are absent. The drug discrimination procedure offers four key advantages for our purpose. First, it is sensitive enough to detect activity at individual pharmacological targets of drugs which, like clozapine, engage multiple receptors. Second, it is an unbiased approach in that it requires no *a priori* knowledge of said targets. Third, it is capable of detecting low doses of drugs, often times lower than those necessary to exert robust effects in other paradigms or even produce detectable levels of drug in blood or cerebrospinal fluid. Finally, and most importantly to the central question, it is applicable to both rats and mice. Using the drug discrimination procedure, we sought to examine whether CNO would produce clozapine-like interoceptive stimulus effects in mice and rats trained to discriminate a low dose of clozapine.

## Results

### Experiment 1: Effects of CNO in Rats and Mice Trained to Discriminate 1.25 mg/kg Clozapine vs. Vehicle

**Figure 1:**
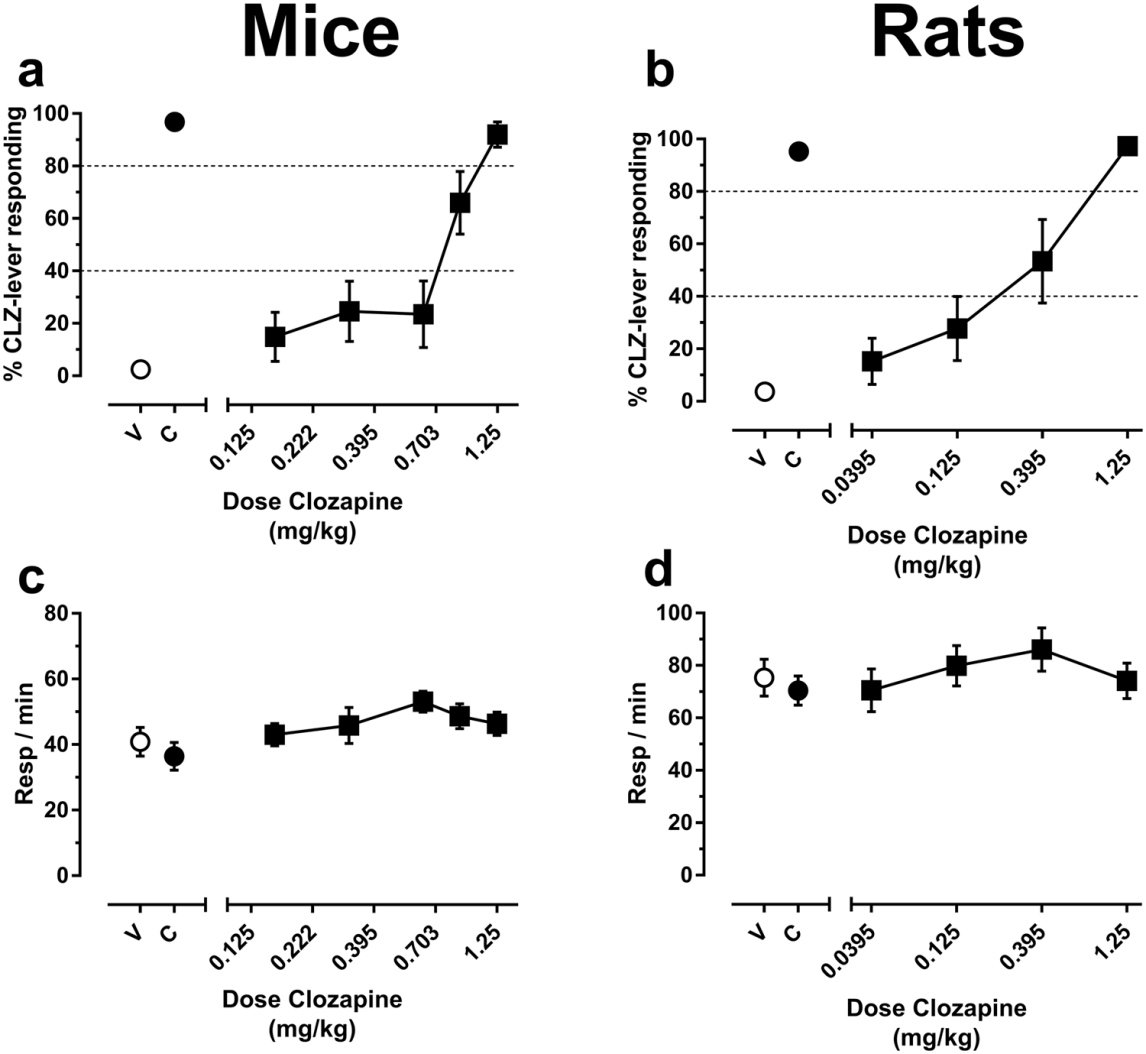
Clozapine functions as a discriminative stimulus in standard laboratory rats and mice. Shown is the mean (±SEM) % clozapine-appropriate responding (%CLZ-lever responding, top panels) or response rates (bottom panels) following substitution tests with clozapine. Data points above “V” and “C” depict averaged data acquired after administration of the clozapine vehicle or the 1.25 mg/kg training dose of clozapine, respectively, during training sessions. (**a**) Clozapine substitution tests in mice. (**b**) Clozapine substitution in rats. Response rates are shown in (**c**) mice and (**d**) rats. Dotted lines in **a** and **b** are used to visually distinguish effect range of no substitution (<40% CLZ-appropriate responding), partial substitution (40–79% CLZ-appropriate responding), or full substitution (≥80% CLZ-appropriate responding). N = 10 per group.

From the start of two-lever training, the mean (±SEM) number of sessions required for mice (n = 10) to demonstrate accurate and stable performance on the clozapine discrimination task was 23.6 (±3.4) sessions, while rats (n = 10) learned the task in 35.6 (±4.1) sessions. As expected, clozapine dose-dependently substituted for its own discriminative stimulus, with partial substitution (40–79% clozapine-appropriate responding) occurring at 0.88 mg/kg for mice (Fig. 1a) and 0.395 mg/kg for rats (Fig. 1b), and full substitution (≥80% clozapine-appropriate responding) occurring at the 1.25 mg/kg training dose in both species. Response rates were not disrupted by any dose of clozapine (Fig. 1c,d), indicating that clozapine did not produce activity-suppressant effects (mice: F_(3,27)_ = 2.07, p = 0.13; rats: F_(5,49)_ = 0.66, p = 0.66).

We further confirmed that the animals had learned to recognize specifically the interoceptive effects of clozapine and not simply any drug-induced state by testing several compounds with varying degrees of similarity to clozapine for their capacity to substitute for the clozapine stimulus. Olanzapine, which is both structurally and pharmacologically similar to clozapine, produced an interoceptive state that fully substituted for the clozapine stimulus in both rats and mice (Table 1). By contrast, compounds with partially-overlapping pharmacological similarity to clozapine (e.g. the α_1_-adrenergic receptor antagonist prazosin, the serotonin 5-HT_2A/2B/2C_ receptor antagonist ritanserin) produced partial substitution, while compounds lacking a shared pharmacological action (e.g. the β-adrenergic receptor antagonist propranolol) failed to substitute (<40% clozapine-appropriate responding) (Table 1). Thus, the animals used in these studies selectively recognized a clozapine-induced interoceptive state and could discern its discrete pharmacological components (detected behaviorally as partial substitution), as has been reported previously^10^.

**Table 1:** Substitution tests in animals trained to discriminate 1.25 mg/kg clozapine vs. vehicle.

	Test drug (mg/kg)	% CLZ-lever resp. (±SEM)	Resp/min (±SEM)	Substitution for CLZ
Rat				
*Training Drugs*	Vehicle	03.68 (0.60)	75.37 (7.10)	—
	Clozapine (1.25)	95.22 (0.57)	70.44 (5.55)	—
*Test Drugs*	Olanzapine (1.0)	84.91 (5.46)	53.28 (6.85)	Full
	Prazosin (0.56)	68.83 (14.62)	54.53 (6.53)	Partial
	Risperidone (0.56)	54.32 (9.32)	28.53 (5.20)	Partial
	Propranolol (10.0)	36.88 (13.81)	58.1 (7.06)	None
Mouse				
*Training Drugs*	Vehicle	02.42 (0.79)	40.86 (4.37)	—
	Clozapine (1.25)	96.83 (1.00)	36.37 (4.26)	—
*Test Drugs*	Olanzapine (0.5)	87.75 (6.19)	49.66 (5.24)	Full
	Prazosin (10.0)	45.14 (17.50)	40.59 (7.06)	Partial
	Ritanserin (16.0)	45.65 (16.72)	44.31 (6.05)	Partial
	Haloperidol (0.1)	17.00 (11.55)	46.59 (1.85)	None

**Figure 2:**
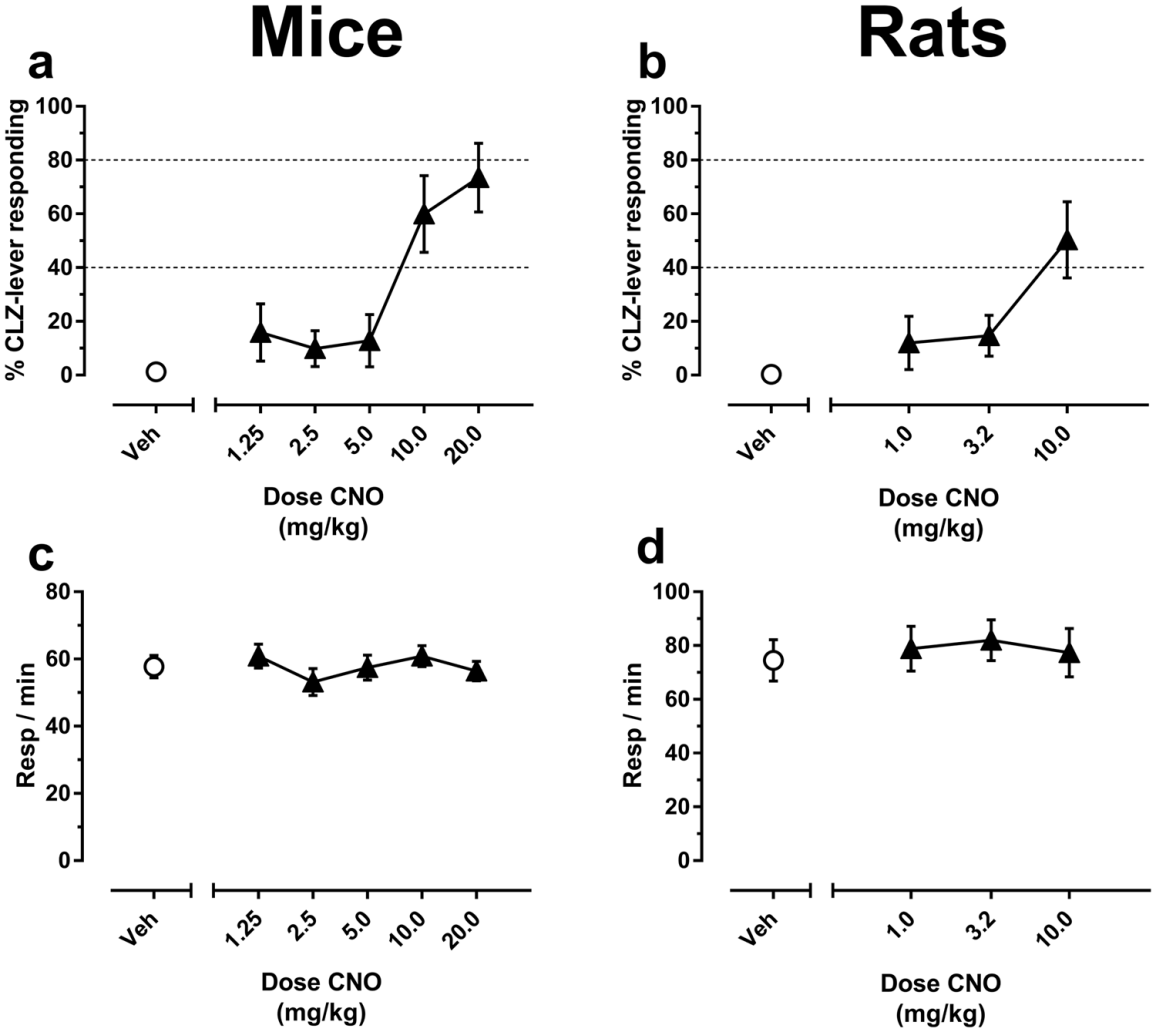
CNO produces clozapine-like interoceptive stimulus effects in standard laboratory rats and mice. Shown is the mean (±SEM) % clozapine-appropriate responding (%CLZ-lever responding, top panels) or response rates (bottom panels) following substitution tests with the CNO vehicle (“Veh”) or several doses of CNO. (**a**) CNO substitution tests in mice. (**b**) CNO substitution tests in rats. Response rates are shown in (**c**) mice and (**d**) rats. Dotted lines in **a** and **b** are used to visually distinguish effect range of no substitution (<40% CLZ-appropriate responding), partial substitution (40–79% CLZ-appropriate responding, or full substitution (≥80% CLZ-appropriate responding). N = 10 per group.

We next asked whether administration of CNO produces a clozapine-like interoceptive stimulus in these animals, using a range of doses that encompassed those typically employed in DREADD studies (up to 20.0 mg/kg in the mouse; up to 10.0 mg/kg in the rat). In the mice, CNO dose-dependently increased allocation of responding to the clozapine-appropriate lever, with high levels of partial substitution occurring at the 10.0 and 20.0 mg/kg doses (Fig. 2a). A similar dose-dependent effect was observed in rats, with 10.0 mg/kg CNO producing partial substitution (Fig. 2b). CNO did not affect response rates at any dose tested (Fig. 2c,d), indicating that it produced clozapine-like effects in the absence of measurable disruptions to baseline operant performance (mice: F_(3,30)_ = 1.15, p = 0.35; rats: F_(2, 20)_ = 0.44, p = 0.68).

At first glance, it appeared that no single dose of CNO produced full substitution in either species. However, because effective doses of CNO varied considerably between animals, we suspected that the detection of full substitution may have been obscured when the data were considered exclusively as a group mean. Inspection of the individual levels of clozapine-appropriate responding at each dose of CNO tested revealed that CNO was indeed capable of producing full substitution in a subset of mice and rats, even at the lower CNO doses which are most commonly used to activate DREADDs (1.0–3.0 mg/kg CNO) (Table 2).

**Table 2:** Substitution of CNO for the 1.25 mg/kg clozapine discriminative stimulus.

CNO dose and pretreatment time	Group mean (±SEM) % clozapine-lever responding	#animals exhibiting partial substitution^1^	#animals exhibiting full substitution^2^
Rat			
1.0 (30 min)	35.60 (10.61)	3/10	1/10
3.2 (30 min)	34.31 (12.12)	2/10	2/10
10.0 (30 min)	31.85 (11.77)	1/10	2/10
1.0 (60 min)	11.99 (09.90)	0/10	1/10
3.2 (60 min)	14.72 (07.68)	0/10	1/10
10.0 (60 min)	50.29 (14.18)*	2/10	4/10
Mouse			
1.25 (30 min)	15.88 (10.65)	1/10	1/10
2.5 (30 min)	09.84 (06.69)	1/10	0/10
5.0 (30 min)	12.79 (09.74)	0/10	1/10
10.0 (30 min)	60.00 (14.30)*	2/10	5/10
20.0 (30 min)	73.49 (12.79)*	1/10	7/10

*Indicates mean effect of partial substitution, averaged across all subjects.

^1^40–79% CLZ-lever responding.

^2^≥80% CLZ-lever responding.

### Experiment 2: Pharmacokinetic Analysis of CNO in Rats and Mice

Plasma levels of clozapine, CNO, and the active clozapine metabolite *N*-desmethylclozapine (NDMC) were quantified in rats and mice following administration of the 1.25 mg/kg training dose of clozapine or 10.0 mg/kg CNO, a dose that both has been used to activate DREADDs and produced moderate (rats) and high (mice) levels of substitution for clozapine in Experiment 1 (Fig. 2a,b).

**Figure 3:**
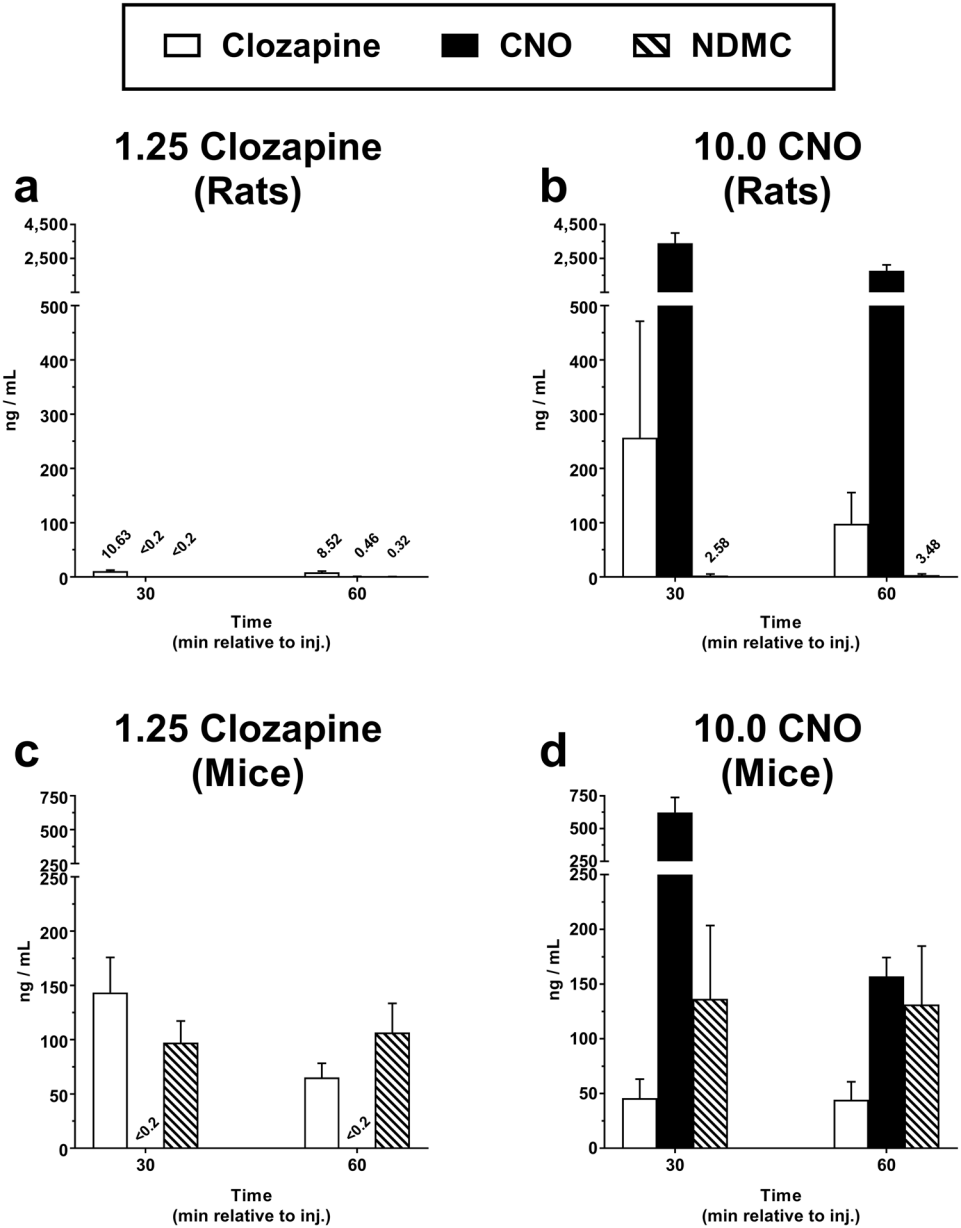
CNO is converted to clozapine in standard laboratory rats and mice. Plasma samples were collected 30 min and 60 min after injection of 1.25 mg/kg clozapine or 10.0 mg/kg CNO and analyzed via UPLC-LC-MS/MS for concentrations of clozapine, CNO, and *N*-desmethylclozapine (NDMC). Data for each analyte are presented as the mean (±SEM) concentration. Mean values are reported above histograms when values were below 15 ng/ml. A value reported as <0.2 ng/ml indicates that the analyte was not present above the limit of detection. Pharmacokinetic analysis following (**a**) clozapine in rats, (**b**) CNO in rats, (**c**) clozapine in mice, and (**d**) CNO in mice. N = 4 per group.

Administration of 1.25 mg/kg clozapine in rats (Fig. 3a) resulted in 10.63 ± 1.99 ng/ml clozapine in plasma 30 min post injection, which decreased only slightly to 8.52 ± 2.12 ng/ml by the 60 min time point. Levels of the two primary metabolites for clozapine (CNO and NDMC) were below limits of detection 30 min post injection, but emerged at very low quantities (<0.5 ng/ml) at 60 min. The ratios of CNO to clozapine and NDMC to clozapine at 60 min post clozapine injection were 5.4% and 3.8%, respectively. In general, the levels of clozapine detected and the pharmacokinetic profile we observed were very similar to those reported previously in the same rat strain using a nearly-identical dose of clozapine (1.0 mg/kg)^11^.

Administration of 10.0 mg/kg CNO to rats resulted in a robust rise in plasma CNO levels (peak at 30 min; 3,404.13 ± 596.84 ng/ml), accompanied by the simultaneous emergence of clozapine at levels far higher than those produced by the training dose of 1.25 mg/kg clozapine (peak at 30 min; 256.73 ± 214.56 ng/ml) (Fig. 3b). The ratio of clozapine to CNO at the 30 min time point in our Sprague-Dawley rats was 7.5%, similar to that reported following 5.0 mg/kg CNO administration in Long-Evans rats (~13%) at the same time point^8^. It was interesting to note that in both studies, the emergence of NDMC increased gradually over a period of time during which levels of clozapine and CNO were decreasing. Furthermore, our calculated NDMC to clozapine ratio at 60 min post CNO administration was 3.6%, which was almost identical to the ratio of 3.8% following administration of clozapine. Combined, these results suggest a pharmacokinetic profile in which systemically-administered CNO is rapidly converted to clozapine in the rat, with the subsequent metabolism of clozapine to CNO and NDMC occurring gradually.

**Figure 4:**
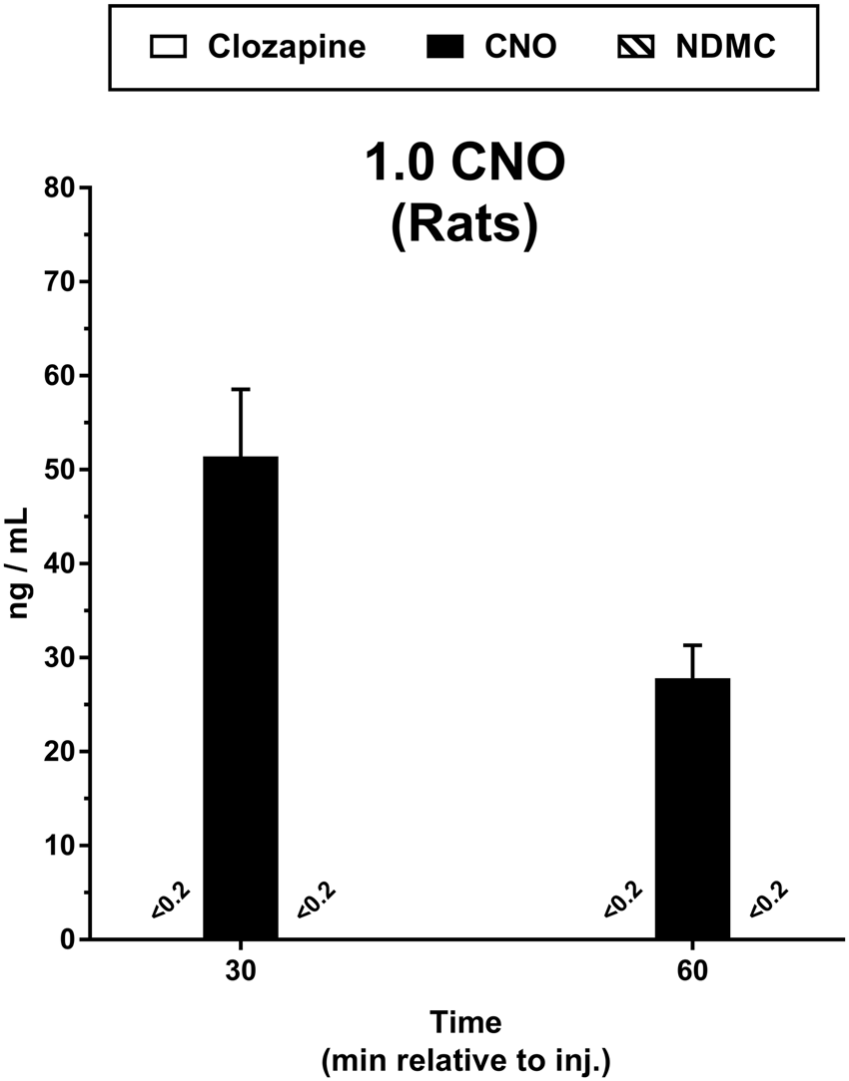
Pharmacokinetic analysis following administration of 1.0 mg/kg CNO i.p. in rats. Plasma samples were collected 30 min and 60 min after injection CNO and analyzed via LC-MS/MS for concentrations of clozapine, CNO, and *N*-desmethylclozapine (NDMC). Data for each analyte are presented as the mean (±SEM) concentration. A value reported as <0.2 ng/ml indicates that the analyte was not present above the limit of detection. N = 4 per group.

While the 10.0 mg/kg CNO dose was selected in this experiment because it engendered maximal average substitution for the clozapine discriminative stimulus, lower doses are more typically employed to activate DREADDs in rats^12^. We therefore quantified levels of CNO, clozapine, and NDMC following administration of 1.0 mg/kg CNO in rats, a dose which is frequently used in DREADD studies and which showed partial-to-full substitution in a small subset of discrimination subjects. Administration of 1.0 mg/kg CNO to rats resulted again in a measurable rise in plasma CNO levels (peak at 30 min; 51.40 ± 7.16 ng/ml) (Fig. 4). In contrast to the higher dose of 10.0 mg/kg however, neither clozapine nor NDMC were detectable in plasma above limits of detection.

Administration of the 1.25 mg/kg training dose of clozapine in mice (Fig. 3c) produced mean clozapine plasma levels of 143.53 ± 32.21 ng/ml 30 min post injection which decreased approximately 2.2-fold to 65.33 ng/ml ± 12.93 by 60 min. NDMC was detectable alongside clozapine and, as was observed in the rat, increased slightly while clozapine levels decreased over time. Interestingly, CNO was not detected following this low-dose clozapine administration, reinforcing previous observations that NDMC is the primary metabolite in this species^13,14^. Administration of 10.0 mg/kg CNO to mice resulted in an expected rise in plasma CNO levels (peak at 30 min; 623.7 ± 114.1 ng/ml) which decreased roughly 5.5 fold by 60 min (Fig. 3d). More important was the detection of high levels of clozapine and NDMC at 30 min post injection (clozapine, 45.9 ng/ml; NDMC, 136.5 ng/ml) and 60 min post injection (clozapine, 44.4 ng/ml; 131.4 ng/ml), demonstrating that CNO unequivocally undergoes significant conversion to clozapine in the mouse. At 30 min post CNO injection, the ratio of clozapine to CNO was 7.4%, which closely resembled the ratio observed in rat at the same time point (7.5%), suggesting that the rate of CNO-to-clozapine conversion is similar across the two species.

## Discussion

The major goals of the present study were to examine the pharmacokinetic profile of CNO in mice and rats and determine whether CNO exerts clozapine-like discriminative stimulus effects in these species in the absence of DREADD expression. Our findings demonstrate that CNO is indeed reverse-metabolized to clozapine in both rats *and* mice, and that doses of CNO commonly used by the scientific community to activate DREADDs are capable of producing an interoceptive stimulus similar to that produced by its parent compound, clozapine. While the results of our pharmacokinetic analysis of CNO in rats are well in line with previous work^7,8^, the results of our pharmacokinetic analysis in mice at first appear to contradict a previous mouse study that reported insignificant plasma levels of clozapine following 1.0 mg/kg CNO administration^9^. However, the scale on the figure provided in that report makes it difficult to discern whether low levels of clozapine may in fact have been produced. Upon request, we were generously provided the raw data from the corresponding author (Jurgen Wess, personal communication), which revealed the detection of 2.78 ng/ml clozapine and 30.17 ng/ml CNO 30 min following CNO administration. The resultant clozapine-to-CNO ratio of 9.21% is close to our measured 7.4% conversion ratio, and is therefore consistent with our current findings rather than incongruent. Taken together, the findings indicate that CNO’s reverse-metabolism to clozapine is a robust and reliable phenomenon in both rats and mice, and that this original report should no longer be cited as evidence that the conversion of CNO to clozapine in mice is negligible.

It is clear based on our results that CNO is rapidly converted to clozapine in the rat (Fig. 3a) and the mouse (Fig. 3c). However, the fate of this converted clozapine shows interspecies differences. In the rat, clozapine is slowly metabolized to both CNO (which can be converted again to clozapine) and NDMC (which is a metabolic endpoint), whereas in the mouse, clozapine is more rapidly metabolized to NDMC, with little evidence of metabolism to CNO. This might be an important distinction given that NDMC is a pharmacologically active metabolite and itself can produce clozapine-like interoceptive stimulus effects^15^. Whether the emergence of higher levels of NDMC in the mouse as compared to the rat plays a functional role in the off-target effects of CNO administration remains to be determined, but it is worth nothing that CNO produced higher overall substitution for the clozapine stimulus in mice in the present study. It is plausible that the mouse-specific emergence of NDMC following CNO administration is at least partially responsible for this species difference.

It was surprising that the levels of clozapine produced by 10.0 mg/kg CNO were dramatically different from those produced by 1.25 mg/kg clozapine itself in the rat, given that this dose of CNO produced some degree of substitution for the 1.25 mg/kg clozapine stimulus in both species. This discrepancy may be related to individual variability in the efficiency of CNO-to-clozapine conversion following CNO administration. For example, rats displayed a range of 13.5–897.8 ng/ml clozapine 30 min post CNO injection, while CNO levels showed a much smaller degree of variability (1681.8–4310.8 ng/ml), suggesting that the variability in emergent clozapine levels was due to differential rates of CNO-to-clozapine conversion and/or clozapine metabolism between individual subjects. This high level of variability in CNO-to-clozapine back-metabolism in rats may also explain why only a small subset of rats trained to discriminate clozapine exhibited partial-to-full substitution for low doses of CNO. It is plausible that those subjects which, for reasons still unclear, are susceptible to more efficient CNO-to-clozapine conversion are consequently more vulnerable to off-target effects of CNO, including clozapine-like interoceptive effects. Further investigation will be needed to more directly assess this hypothesis.

A critical observation derived from our collective results is that resultant plasma levels of converted clozapine are *not* predictive of clozapine-like discriminative stimulus effects produced by CNO. Consistent with this supposition, lowering the dose of CNO to 1.0 mg/kg in the rat did not produce detectable plasma levels of clozapine or NDMC in any subject (Fig. 4), but was recognized as clozapine-like in a subset of animals (Table 2) and has reliably been used to trigger DREADD-mediated behavioral effects in other studies^12^. It has been suggested that clozapine efficiently crosses the blood-brain barrier and is sequestered within the brain compartment^11,16^, which may explain why low doses of CNO can produce clozapine-mediated effects in the absence of quantifiable clozapine in blood.

In sum, our results show that systemic administration of CNO at doses used in DREADD studies produces clozapine-like interoceptive stimulus effects in both rats and mice with substantial between-subject variability in the effective dose range. Our pharmacokinetic data suggest that this effect of CNO is mediated via back-conversion to clozapine, a phenomenon which we have confirmed from previous studies in rat and conclusively report in mice for the first time. Moreover, we show that CNO can exert clozapine-like behavior at doses that do not produce measurable levels of clozapine in plasma. In light of all accumulating evidence, researchers employing DREADD technologies should be aware that CNO is *not* an inert substance as initially purported in rats and mice. However, we do not believe that these limitations abolish the utility of DREADDs. Rather, like most neuroscience tools, DREADDs are not perfect, and we strongly advocate for the exercise of appropriate caution when designing studies employing them. While it has already been suggested that CNO should be administered to non-DREADD-expressing animals to control for off-target activity^8^, we would expand this recommendation to include close scrutiny in individual subjects, specifically comparing CNO injection to vehicle injection when possible within the same subject. Our present results also provide important insights for the identification and characterization of novel DREADD ligands, especially those that are structurally related to clozapine and CNO. Given that the drug discrimination assay was sensitive enough in the present study to detect active doses of CNO that failed to produce measurable levels of clozapine in plasma (i.e. 1.0 mg/kg CNO in the rat), we suggest that any potential DREADD agonist (e.g. “compound 21”) derived from the CNO structure^17^ should be screened in animals trained to discriminate low-dose clozapine vs. vehicle, regardless of whether the compound is suspected of or has demonstrable conversion to clozapine. This vital role for the drug discrimination assay is perhaps best exemplified when considering perlapine, a clinically-approved compound which was recently reported to exhibit potent and selective activation of the hM3Dq DREADD^17^. As a result of this discovery, perlapine is now being marketed and sold by a number of popular suppliers of research compounds as a novel and selective DREADD agonist. However, there is a general lack of acknowledgement that perlapine has been found to fully substitute for clozapine’s interoceptive stimulus effects in nonhuman primates^18^. We therefore propose that the drug discrimination procedure should be a requisite methodological tool with which to further characterize CNO, clozapine, perlapine, compound 21, and other potential DREADD agonists, and can even be expanded to encompass other chemogenetic approaches such as the κ-opioid-derived DREADD-salvinorin B system^19,20^. The goal of such studies moving forward will be to identify doses of these compounds that activate DREADDs in DREADD-expressing animals, but do not produce interoceptive stimulus effects in either DREADD-expressing subjects or their non-DREADD-expressing counterparts.

## Materials and Methods

### Subjects

Ten adult male Sprague-Dawley rats (Charles River Laboratories Inc., Wilmington, MA, USA) weighing approximately 250–450 g over the duration of the study served as subjects for drug discrimination experiments. A separate cohort of eight adult male Sprague-Dawley rats (Charles River Laboratories Inc.) served as subjects for the pharmacokinetic studies. Rats were individually housed in a climate-controlled room under a reverse 12-h light/dark cycle (lights on 2000 to 0800). The rats serving in drug discrimination experiments were maintained at ~90% free-feeding weight by providing 16–18 g of standard rodent chow daily approximately 30–60 min following training/test sessions, while the eight rats employed in pharmacokinetic studies were provided food *ad libitum* in the home cage throughout the duration of experiments. Water was available *ad libitum* to all rats in their home cage. Behavioral experiments were conducted 5–6 days/week in operant chambers located within the vivarium between the hours of 1300 and 1600.

Ten adult B6129 inbred mice (male, n = 7; female, n = 3) weighing between 20–30 g (Harlan Laboratories, Indianapolis, IN, USA) served as subjects in drug discrimination experiments. These mice were individually housed in clear plastic cages within a climate-controlled vivarium on a 12 h light/dark cycle (0600/1800 and maintained at 85–90% free-feeding body weights on standard rodent chow which was made available in the home cage ~30 min after daily training/testing for the duration of the study. Water was available *ad libitum* in home cages. Mice were moved daily (6 to 7 days each week) from the vivarium to the laboratory where testing occurred.

A separate cohort of twenty adult male B6129 inbred mice (Taconic Biosciences, Germantown, NY, USA) weighing 25–35 g at the time of study served as subjects for pharmacokinetic analyses. These mice were group-housed in standard polycarbonate cages and had *ad libitum* access to rodent chow and water. All studies were conducted in strict accordance with the National Institutes of Health’s “Guide for Care and Use of Laboratory Animals” and were approved by the Institutional Animal Care and Use Committee at Emory University or the Institutional Animal Care and Use Committee at Virginia Commonwealth University.

### Drug discrimination procedure

Rats and mice were trained to discriminate 1.25 mg/kg clozapine from its vehicle using a two-lever, food-reinforced drug discrimination procedure based on methods published previously^15,21–23^ and described in detail in the Supplementary Materials and Methods. Briefly, each animal was assigned one lever as the “clozapine-appropriate lever”, and the other lever as the “vehicle-appropriate lever.” When the subject was injected with clozapine, only responses on the “clozapine-appropriate lever” were reinforced, whereas only responses on the “vehicle-appropriate lever” were reinforced following injection of vehicle. Animals received only one injection per day. The 1.25 mg/kg dose of clozapine was selected as the training dose in these studies because this is the lowest dose of clozapine that has been reported to function as a discriminative stimulus^22,23^.

Substitution tests occurred only when animals had satisfied strict performance criteria (see Supplementary Methods and Materials). To confirm the selectivity of the clozapine stimulus and its control over behavioral responding, rats were tested with vehicle or multiple doses of clozapine (0.0395, 0.125, 0.395, 1.25 mg/kg), the mixed dopamine/serotonin/norepinephrine antagonists olanzapine (1.0 mg/kg) and risperidone (0.56 mg/kg), the α_1_-adrenergic receptor antagonist prazosin (0.56 mg/kg), and the β-adrenergic receptor antagonist propranolol (10.0 mg/kg). Similarly, mice were tested with vehicle and multiple doses of clozapine (0.156, 0.3125, 0.625, 0.88, 1.25 mg/kg), olanzapine (0.5 mg/kg), the nonselective serotonin 5-HT_2_ receptor antagonist ritanserin (16.0 mg/kg), prazosin (10.0 mg/kg), and the nonselective dopamine D_2_-like receptor antagonist haloperidol (0.1 mg/kg). The doses of olanzapine, ritanserin, prazosin, and haloperidol were used because we have observed that higher doses produce nonspecific rate-suppressant effects. To test for CNO-induced clozapine-like effects, animals were administered CNO (rats −1.0, 3.2, 10.0 mg/kg; mice −1.25, 2.5, 5.0, 10.0, 20.0 mg/kg) prior to a test session. All drugs and doses were administered in a randomized order to each subject.

### Blood Sample Collection and Analysis

Rats were surgically prepared with chronic indwelling intrajugular catheters as described previously^24^ to allow for rapid and repeated blood sampling. Blood collections began at least two weeks following surgery. On a test day, rats were administered either clozapine (1.25 mg/kg) or CNO (1.0, 10.0 mg/kg) and returned to the home cage. Blood samples (0.4–0.5 ml per sample, ~0.1 ml withdrawn per 5 s) were collected via aspiration from the intravenous catheter 30 and 60 min following drug administration. Catheters were flushed with bacteriostatic saline and locked with 0.1 ml of heparinized saline (300 heparin IU/ml) when not in use to maintain catheter patency between collections and on days between tests. Tests were separated by a minimum of 2 weeks and were performed in the following order for all subjects: (1) 10.0 mg/kg CNO, (2) 1.25 mg/kg clozapine, (3) 1.0 mg/kg CNO.

Mice were administered either clozapine (1.25 mg/kg) or CNO (10.0 mg/kg) and returned to their home cage. 2–3 min prior to the desired time point of blood sampling, each mouse was placed in a Plexiglas anesthesia induction chamber and exposed to 4–5% isoflurane until loss of movement and then transferred to a nosecone that continued to supply isoflurane (1–2%). Once deep anesthesia was verified, the heart was exposed and a 23 g needle attached to a 1 ml syringe was inserted into the left ventricle. 0.4–0.5 ml of blood was withdrawn and handled identical to the description above for rat blood sample collections.

All blood samples were deposited into a 1.7 ml tube containing 10 μl of heparinized saline (500 heparin IU/ml) and stored on ice until centrifugation at room temperature at 800 g for 10 min. The plasma was then removed, placed into a separate sterile 1.7 ml tube, and stored at −80 °C until subsequent analysis via UPLC-LC/MS/MS (see Supplementary Materials and Methods).

### Drugs

Clozapine was provided as a generous gift to J.H.P. from Novartis (Hanover, NJ, USA). Olanzapine was provided as a generous gift to J.H.P. from Eli Lilly (Indianapolis, IN, USA). Clozapine, olanzapine, and risperidone were supplied to D.W. by the National Institute of Mental Health’s Chemical Synthesis and Drug Supply Program. Haloperidol, prazosin, propranolol, and ritanserin were purchased from Sigma-Aldrich (St. Louis, MO, USA). J.H.P. and M.S.F. obtained CNO from the Rapid Access to Investigative Drug Program funded by the National Institute of Neurological Disorders and Stroke. D.W. obtained CNO from the National Institute on Drug Abuse Drug Supply Program.

Clozapine, olanzapine, risperidone, haloperidol, prazosin, propranolol, and ritanserin were each dissolved in distilled water with 2–3 drops of lactic acid and pH-adjusted to 6.0–7.0 with NaOH. For mouse drug discrimination studies, CNO was also dissolved in this vehicle. For rat drug discrimination studies and for mouse and rat pharmacokinetic analyses, CNO was dissolved in bacteriostatic saline containing v/v 2.5–5.0% dimethyl sulfoxide (Sigma-Aldrich) and 10% Cremophor EL (Sigma-Aldrich).

For mouse drug discrimination studies, all drugs were administered s.c. at a volume of 10 ml/kg, 30 min prior to session onset. For rat drug discrimination studies, all drugs were administered i.p. at a volume of 1 ml/kg. Clozapine was administered 60 min prior to session onset, while olanzapine, risperidone, prazosin, and propranolol were administered 30 min prior to session onset. CNO was tested at both 30 and 60 min pretreatment times. All drug doses are expressed as the salt weight.

### Data analysis

For drug discrimination studies, % clozapine-lever responding was calculated as the number of lever presses emitted on the clozapine-appropriate lever divided by the total number of lever presses on both levers multiplied by 100. Classification of substitution for the 1.25 mg/kg clozapine stimulus was designated as follows: <40% clozapine-lever responding, no substitution; 40–79% clozapine-lever responding, partial substitution; ≥80% clozapine-lever responding, full substitution. Response rates were calculated by dividing the total number of lever presses by the total run time in minutes. Response rates were compared using repeated-measures one-way analysis of variance (ANOVA) with one exception: response rates from clozapine substitution studies in rats were analyzed using an independent one-way ANOVA because the 0.0395 and 0.395 mg/kg clozapine doses were tested in 6/10 and 9/10 subjects, respectively. Figures were plotted and statistical analyses performed using GraphPad Prism v 7.3 (GraphPad Software Inc., La Jolla, CA, USA). For all statistical analyses, significant differences were accepted at the 95% level of confidence (α = 0.05).

### Data availability

The data that support the findings of this study are available from the corresponding author upon reasonable request.

## Electronic supplementary material

Supplementary Info
