# Supplementary material for: The DREADD agonist clozapine N-oxide (CNO) is reverse-metabolized to clozapine and produces clozapine-like interoceptive stimulus effects in rats and mice
Source: Sci Rep. 2018 Mar 1;8:3840. doi: 10.1038/s41598-018-22116-z (PMC5832819; doi:10.1038/s41598-018-22116-z)
Supplement: Supplementary file 1 — Supplementary Info [file 41598_2018_22116_MOESM1_ESM.pdf]

<u>Supplementary Information:</u>

The DREADD agonist clozapine *N*-oxide (CNO) is reverse-metabolized to clozapine and produces clozapine-like interoceptive stimulus effects in rats and mice

Daniel F. Manvich Ph.D., Kevin A. Webster Ph.D., Stephanie L. Foster B.S., Martilias S. Farrell Ph.D., James C. Ritchie Ph.D., Joseph H. Porter Ph.D., and David Weinshenker Ph.D.

Supplementary Materials and Methods:

Drug Discrimination Training and Testing: Rats

Behavioral sessions were conducted in rodent operant chambers located within light- and sound-attenuating enclosures (Med-Associates Inc., St. Albans, VT). Each chamber contained an operant panel equipped with two retractable levers, a white stimulus light located above each lever, and a receptacle for food-pellet delivery centered between the two operant levers. A house light was mounted to the chamber wall opposite of the operant panel, and a food hopper was mounted behind the operant panel that delivered 45 mg food-pellets (F0165, Bio-Serv, Flemington, NJ) to the food receptacle via a plastic tubing connection. A fan was secured to the enclosure wall to provide constant air flow throughout behavioral sessions and to further reduce the influence of ambient noise. Med-PC IV software (Med-Associates Inc.) was interfaced with each chamber to allow for automated output control and lever-press recording.

Subjects were first trained 5 days/week to lever-press for food delivery using a fixed ratio (FR) schedule of reinforcement. The session began with extension of both levers into the operant chamber and the illumination of a stimulus light above each lever to signal reinforcer availability. A single lever-press on either lever (FR1) resulted in delivery of a single 45 mg food pellet, retraction of the lever on which the response was emitted, the termination of both stimulus lights, and illumination of the house light for 5 s, during which responses on the other lever had no scheduled consequences. Following this 5 s timeout (TO), the house light was extinguished, both stimulus lights were illuminated, and a single response on the remaining lever resulted in reinforcement and TO as described above. Both levers were then re-extended and sessions continued in this manner until 1 h elapsed or 60 reinforcers were earned,

whichever occurred first. The FR requirement was increased by 1 every 20th reinforcer and carried over across sessions. When the FR was ≥ 2, responses on one lever reset the ratio of the opposite lever to 0, and therefore consecutive responses were required to satisfy an FR requirement. This phase of training continued until rats performed stably (i.e. earned all 60 reinforcers and response rates varied by < 20%) across 3 consecutive sessions under an FR10 schedule of reinforcement.

Next, each rat was trained in daily sessions to discriminate clozapine (1.25 mg/kg i.p.) from its vehicle (1 ml/kg, i.p.). Animals received only one injection per day. Each session began with the administration of either clozapine or vehicle, and the rat was then immediately placed into the operant chamber. For the next 60 min, all stimulus lights were turned off and the response levers were retracted. Once this 60 min start delay had elapsed, the stimulus lights above each lever were illuminated and both levers were extended to signal the start of the session and food availability. During this phase of training, the completion of 10 consecutive responses on the injection-appropriate lever (FR10) was reinforced by a 45 mg food pellet. The delivery of the food pellet was paired with the illumination of the house light and termination of the stimulus lights for 5 sec (lever-presses were not reinforced during this TO). Responses on the injection-inappropriate lever reset the ratio on the injection-appropriate lever but otherwise had no consequence. Lever assignments (i.e. clozapine-appropriate lever vs. vehicle-appropriate lever) were counterbalanced among subjects. Each daily session was terminated once the rat had earned 20 reinforcers or 15 min elapsed, whichever occurred first. Clozapine and its vehicle were initially administered in a double-alternation schedule (i.e. clozapine, clozapine, vehicle, vehicle, clozapine, clozapine, vehicle, vehicle, …), with only one injection per

day. This phase of training continued until the following criteria were met for five consecutive sessions: 1) ≥ 80% of responses for the first reinforcer were emitted on the injection-appropriate lever and 2) ≥ 80% of the total responses across the entire session were emitted on the injection-appropriate lever. Once rats had successfully learned the drug discrimination task, the schedule of injection presentations was switched to a pseudorandomized schedule for the remainder of the study. During the pseudorandom schedule, animals never received the clozapine training dose for more than 3 days in a row, nor did they receive more than 4 total clozapine doses per week. The schedule was designed to prevent the accumulation of clozapine or any of its active metabolites from impacting responding on any given day. We (unpublished results) and others (Baldessarini *et al*, 1993) have found that clozapine and its metabolites are no longer measurable in blood or brain 24 hours following administration. Substitution tests began when rats again satisfied the aforementioned criteria for five consecutive sessions on the pseudorandomized schedule.

Substitution tests occurred only when animals had satisfied the criteria described above in 5/5 or at least 6/7 previous consecutive training sessions. Substitution test sessions were conducted identical to training sessions with a few important exceptions. First, in some instances, the start delay was reduced to 30 min where indicated. Second, completion of an FR10 on either lever was reinforced. Finally, responses on one lever did not reset the ratio on the other lever, and thus consecutive responses were not required for reinforcer delivery.

Drug Discrimination Training and Testing: Mice

Drug discrimination experiments were conducted in six computer-interfaced mouse operant conditioning chambers (Med Associates Inc.), with two retractable levers positioned on the left and right positions equidistant on the front wall. A recessed well in which a liquid dipper delivered 0.02 ml of sweetened milk (by volume: 150 ml sugar, 150 ml powdered non-fat milk, 500 ml water) was positioned between the two levers, and a house light was mounted on the opposite wall. Operant chambers were housed in sound- and light-attenuating cubicles (Med Associates Inc.). Experimental events and data collection during these experiments were controlled by Med-PC software (Med Associates Inc., version 1.17).

Mice were initially acclimated to the sweetened condensed milk in the home cage for two days and then received noncontingent presentation of the sweetened milk reinforcer in the operant test chambers in two 15-min sessions. Mice were then trained to lever press on a single lever (the vehicle-appropriate lever, counterbalanced across animals) on a FR1 schedule of reinforcement. Each training session began with illumination of the house light (illuminated throughout session) and extension of one or both levers into the chamber, depending on training condition (see below). Initially, a single lever press (FR1) resulted in the delivery of 0.02 ml sweetened milk, which was available to the mouse in a raised dipper cup for 3 s before being withdrawn. The FR requirement was gradually increased until all mice were responding under a FR10 schedule of reinforcement and response rates reached levels consistently higher than 10 responses/min. Mice were then injected daily with vehicle 30 min prior to each training session. Only the vehicle-appropriate lever was extended into the operant chamber and responding was reinforced on a FR10 schedule. Once response rates again stabilized at >10 resp/min, mice were

injected with 2.5 mg/kg clozapine prior to the training session, and only the clozapine-appropriate lever was extended into the chamber, with responses again reinforced according to a FR10 schedule. After 8 days of training with 2.5 mg/kg clozapine, the training dose was lowered to 1.25 mg/kg and held at this dose for the remainder of the study. Once baseline rates stabilized at >10 responses/min, two-lever drug discrimination training began with both levers present during training sessions. The mice received 1.25 mg/kg clozapine or vehicle injections, one injection per day, according to a double alternation schedule (i.e. clozapine, clozapine, vehicle, vehicle, clozapine, clozapine, vehicle, vehicle, …). During two-lever training, only injection-appropriate lever responses were reinforced and an incorrect lever response reset the FR counter. Before substitution testing could begin, mice had to meet the following criteria for at least 5/6 consecutive double-alternation training sessions: 1) > 50% injection-appropriate lever responding for the first reinforcer of the session, 2) ≥ 80% injection-appropriate lever responding across the entire session, and 3) rate of responding ≥ 10 resp/min.

Subjects were required to meet training criteria on consecutive vehicle and clozapine training sessions before substitution testing; this ensured that animals had at least two days between test sessions. Control tests with 1.25 mg/kg clozapine and vehicle were established before testing each new drug. During control and drug substitution test sessions, both levers were presented and responding was reinforced on both levers; however, if an animal switched to the opposite lever before completing the FR10 requirement, the FR counter was reset.

Pharmacokinetic Analysis

Levels of clozapine, CNO, and N-desmethylclozapine were quantified via UPLC-LC/MS/MS. Briefly, sample extraction was accomplished using a standard protein crash (0.1 ml of sample + 50 ml of 0.4M zinc sulphate and 0.2 ml of the internal standards in methanol). The internal standard was d^8-clozapine. The assay was performed on an Acquity UPLC System with a Triple Quadrapole Detector (TQD) in the MRM mode employing ESI positive ionization (Waters Inc., Milford, MA, USA). The mobile phases consisted of (A) 2mM ammonium acetate and 0.1% formic acid in water and (B) 2mM ammonium acetate and 0.1% formic acid in methanol. The flow rate was 0.6 ml/min and the chromatography was developed using a gradient from 25% B to 75% B over 3.5 min on an Acquity UPLC, C-18 column (1.7 µM, 2.1 x 50 mm). 10 µL of extract was injected. The order of elution was N-desmethylclozapine (313 > 269.9), clozapine (327.0 > 270.0), and clozapine N-oxide (342.8 > 243.0, primary transitions). A seven-point standard curve (500 pg/ml to 7.8 ng/ml) was prepared from 1 mg/ml solutions of each analyte supplied by Cerilliant (Round Rock, TX, USA) or Sigma-Aldrich (St. Louis, MO, USA), and two levels of quality control were processed in each run. This method is linear from 0.2 to 2000 ng/ml. The limit of detection was 0.05 ng/ml for all compounds except clozapine N-oxide (0.1 ng/ml), and the limit of quantitation was 0.2 ng/ml for both N-desmethylclozapine and clozapine, and 0.5 ng/ml for clozapine N-oxide (15%). This method exhibits no matrix effects by established criteria (Matuszewski *et al*, 2003). Absolute recoveries ranged from 88.9% to 109.3% and inter-assay imprecisions ranged from 3 to 13% at levels of 75 and 300 ng/ml for all compounds.

References:

Baldessarini, R.J., Centorrino, F., Flood, J.G., Volpicelli, S.A., Huston-Lyons, D., Cohen, B.M. Tissue concentrations of clozapine and its metabolites in the rat. *Neuropsychopharmacology* **9,** 117-124 (1993).

Matuszewski, B.K., Constanzer, M.L., Chavez-Eng, C.M. Strategies for the assessment of matrix effect in quantitative bioanalytical methods based on HPLC-MS/MS. *Anal. Chem.* **75**, 3019-3030 (2003).
